# Analyzing Beach Recreationists’ Preferences for the Reduction of Jellyfish Blooms: Economic Results from a Stated-Choice Experiment in Catalonia, Spain

**DOI:** 10.1371/journal.pone.0126681

**Published:** 2015-06-08

**Authors:** Paulo A. L. D. Nunes, Maria L. Loureiro, Laia Piñol, Sergio Sastre, Louinord Voltaire, Antonio Canepa

**Affiliations:** 1 Ecosystem Services Economics Unit, Division of Environmental Policy Implementation, United Nations Environment Programme (UNEP), United Nations Avenue, 00100, Nairobi, Kenya; 2 Departamento de Fundamentos da Análise Económica, Facultade de Ciencias Económicas, Universidade de Santiago de Compostela, 15782 Campus Norte, Avda Burgo das Nacións, 15704, Santiago de Compostela, Spain; 3 LEITAT Technological Centre - Maritime Division, 2 Carrer de la Innovació, 08225, Terrassa, Spain; 4 Institut de Ciencies del Mar - CSIC, 37-49 Passeig Marítim de la Barceloneta, 08013, Barcelona, Spain; 5 UMR-AMURE, Université de Bretagne Occidentale (UBO), 12 rue de Kergoat, 29200, Brest, France; The Evergreen State College, UNITED STATES

## Abstract

Jellyfish outbreaks and their consequences appear to be on the increase around the world, and are becoming particularly relevant in the Mediterranean. No previous studies have quantified tourism losses caused by jellyfish outbreaks. We used a stated-choice questionnaire and a Random Utility Model to estimate the amount of time respondents would be willing to add to their journey, in terms of reported extra travel time, in order to reduce the risk of encountering jellyfish blooms in the Catalan coast. The estimation results indicated that the respondents were willing to spend on average an additional 23.8% of their travel time to enjoy beach recreation in areas with a lower risk of jellyfish blooms. Using as a reference the opportunity cost of time, we found that the subsample of individuals who made a trade-off between the disutility generated by travelling longer in order to lower the risk of jellyfish blooms, and the utility gained from reducing this risk, are willing to pay on average €3.20 per beach visit. This estimate, combined with the respondents’ mean income, yielded annual economic gains associated with reduction of jellyfish blooms on the Catalan coast around €422.57 million, or about 11.95% of the tourism expenditures in 2012. From a policy-making perspective, this study confirms the importance of the economic impacts of jellyfish blooms and the need for mitigation strategies. In particular, providing daily information using social media applications or other technical devices may reduce these social costs. The current lack of knowledge about jellyfish suggests that providing this information to beach recreationists may be a substantially effective policy instrument for minimising the impact of jellyfish blooms.

## Introduction

Although jellyfish are a natural feature of the Mediterranean, jellyfish blooms are recurring events that are an inconvenience for swimmers, with certain species being a significant health hazard [1]. Media coverage has increased people’s awareness about jellyfish blooms [2], particularly on the Catalan coast for the period 1980–2012 (reviewed in [3]. Jellyfish are considered detrimental to fish and fishing because they clog fishing nets, drive away fish and consume fish eggs and larvae [4], [5]. The resulting socio-economic impacts, both direct (tourism) and indirect (coastal development, fisheries), have become tangible and significant. Although overfishing, coastal habitat degradation and climate change are amongst the most probable drivers [6], we still lack sufficient information on the economic impacts and consequences of these blooms.

Very few studies report the impacts of jellyfish blooms on the economic sectors. Some estimates exist in fisheries, such as the Gulf of Mexico shrimp fishery [7], the Black Sea anchovy fishery [8], the Peruvian anchovy fishery [9], multiple fisheries in Korea [10] and Slovenia [11]. Kim et al [10] estimated that the impacts of jellyfish blooms on Korea’s various fisheries ranged between 2.1% and 25% of the total value of the catches. However, jellyfish blooms have many more consequences than those suffered by the fishing fleet, including potentially severe losses in the recreational sectors. Economic valuation of tourism and recreational losses due to jellyfish blooms is very limited or non-existent. The present paper addresses this gap and constitutes the first econometric valuation study of both market and non-market losses.

Similar exercises regarding recreational ecosystem services assessment based on questionnaires have been carried out worldwide. For instance, Hearne and Salinas [12] evaluated several management options in the context of protected areas in Costa Rica. Nunes andVan den Bergh [13] and Nunes and Markandya [14] illustrated the use of alternative, non-market valuation methods to estimate the economic value of the social damage caused by marine bio-invasions on beach recreationists. Underlying economic value assessments were shown to be relevant in undertaking cost-benefit analyses and supported the selection of a policy management practice, including a ballast water treatment plant in the harbour of Rotterdam. In addition, Beaumont et al [15] assessed the goods and services resulting from marine biodiversity in the UK. The present exercise also constitutes a valid tool for informing the general public; Remoundou et al [16] gives a more comprehensive review on non-market valuation to estimate marine ecosystem services.

Therefore, the objective of this paper is to assess the impact of jellyfish blooms on beach recreationists’ preferences in Catalonia (Spain) and their consequent impact on welfare losses in the tourism sector. Catalonia is a region in north-eastern Spain with 580 km of coastline and is a leading coastal tourist destination. In 2012, 263.7 million recreational beach visits were reported [17]. We tested the impact of the risk of jellyfish blooms on beach recreationists by computing the implicit value of the additional time that visitors were willing to travel in order to reduce the probability of encountering a jellyfish bloom at the beach.

## Materials and Methods

### Ethics Statement

Ethical approval was not required for this study, due to the fact that respondents gave their oral consent, responses were kept anonymously, and data on health was not requested. The Spanish Organic Law 15/1999 (December 13) [18], on the Protection of Personal Data excludes these types of studies and datasets from further regulations and administrative procedures.

### Survey design and implementation

The 221 beaches along the coast of Catalonia were classified according to a set of parameters including the municipality/location, the beach area, the water quality/visibility, the level of occupancy/congestion, and the type of the surrounding terrestrial environment (ranging from urban to natural). The combination of these elements allowed us to profile the beaches and helped to select beaches that are representative for the entire Catalan coastline for our study (Table 1). This high degree of representativeness was a relevant objective in order to facilitate the interviewing process in an environment whose results could be extrapolated to the entire Catalonian coast.

**Table 1:** Beaches in Catalonia, Spain analyzed in the study (from SW to NE).

Beach name	Environment	Width (m)	Length (m)	Area (m²)	Jellyfish risk* (%)	Blue flag
Barceloneta	Urban	40	600	24,000	7.07	Yes
De Bogatell	Urban	32	625	18,676	12.96	Yes
De Sabanell	Mixed	32	2,380	75,921	0.75	Yes
De Blanes	Urban	44	610	26,170	0.37	Yes
Gran de Palamòs	Urban	55	990	48,650	9.52	No
De la Fosca	Mixed	37	514	16,461	3.96	Yes
El Castell	Natural	63	339	22,715	7.14	No
El Golfet	Natural	17	75	1,277	5.58	No

* This variable was provided by the Catalan Water Agency and is constructed taking into account the observations of jellyfish blooms with respect to the number of inspections carried out between 2006 and 2010. [Risk = (No. of observations of jellyfish / No. of Inspections)*100]

The beaches selected had different profiles and, therefore, could attract different users or consumers of beach recreation activities, as well as different uses of the beach. Table 1 represents the variety of beaches included in the analysis. For example, Barceloneta is a semi-closed urban beach located in a highly touristic neighbourhood in Barcelona with a high occupation level. Bogatell is a closed urban beach located in the urban area of Barcelona and highly occupied. Sabanell is a semi-closed beach located in a semi-urbanized area in Blanes. Blanes is a closed urban beach located in the city of Blanes. Gran de Palamós is a semi-closed beach located in the urban centre of Palamós with large numbers of visitors, but a low occupation due to its large extent. Fosca is a closed beach located in La Fosca neighborhood in Palamós with a high occupancy level. Castell is a closed natural beach located between the municipalities of Palamós and Palafrurgell, inside the Castell-Cap Roig natural area protected by the Plan for Spaces of Natural Interest. Golfet is a closed natural beach also located in the Castell-Cap Roig area, next to a low urbanized area south of Calella de Palafrurgell, highly occupied due to its small area.

In addition to the sampling of the beaches, we drafted and tested the survey protocol by means of several focus groups and a pilot experiment carried out on the beach. Those activities allowed us to optimize the wording, the use of visual aids and the overall understanding of the final questionnaire by the respondents. In addition, the interviewers received a training session at the Institute of Marine Sciences of the Spanish National Research Council (Institut de Ciències del Mar, CSIC), in order to prepare them for the face-to-face survey. Each interviewer was debriefed about the nature and the objective of the questionnaire and given a toolbox for this operation, including a set of *verbatim* explanations of the questions. At this stage, the final questionnaire was ready for execution.

The final questionnaire contained six sections (S1 Survey). The first questions focused on profiling the respondent with respect to the set of recreational activities conducted on the beach, the size and the composition of people in their group of visitors, the number of the days spent on the beach during the 2011 summer season, the distance travelled to the beach, and a set of questions about the importance attached to recreational activities and other features in choosing the beach. The second section requested information regarding the travel cost and expenditure incurred as a result of visiting the beach (e.g. travel cost and travel time, accommodation cost on that day, meal cost on that day). Respondents were also asked to report the means of transportation they used to get to the beach, including walking. Among the non-residents, information was also collected on the respondents’ type of accommodation, including their holiday home, hotel or whether they were staying with friends/relatives. The third section focused on eliciting the socio-economic impacts of jellyfish blooms, including profiling the respondent’s experience with respect to jellyfish stings and the potential economic cost of treatment.

In the fourth section, respondents faced the stated choice exercise or a choice experiment (CE). For this task, ten multiple-choice cards consisting of three different options were presented to each respondent. The first two options, showed two beaches with different recreational opportunities. The third, as recommended by Adamowicz et al [19], was a non-choice option offering alternatives that included the opportunity to stay at home or do any recreational activity other than going to the beach. It was presented to participants because this is an obvious element of choice behaviour. Recreational opportunities were characterized by four main attributes of the choice: (1) the risk of jellyfish bloom at the beach (measured in the number of days per week in which blooms may happen), (2) the water quality of the beach (related to water purity and transparency), (3) the infrastructure and services available at the beach (coffee shops, toilets, restaurants, etc), (4) the proposed additional travel time (with respect to the reported travel time to reach the beach) that the respondent would incur to reach the beach being considered (Table 2).

**Table 2:** Description of attributes and levels considered for the choice experiment cards administered at beaches in Catalonia, Spain.

Attributes	Levels		
Jellyfish risk bloom	Low risk (≤2days/week)	High risk (> 5 days/week)	
Water transparency	Average (as regulated by the law)	Above average	
Services	Parking and toilets	Parking, toilets and children play area	Parking, toilets, children’s play area and first aid centre
Additional travel time	+5%	+10%	+15%

Respondents were presented with a given stated choice question. These attributes were selected based on the focus groups and preferences towards relevant beach elements in Catalonia.

The first two attributes had two levels, while the rest had three levels. Attributes and their levels were selected from discussions with marine experts in Catalonia, focus groups and pilot studies. The total number of beach profiles generated was 2^2^×3^2^ = 36. To reduce the cognitive burden for respondents, fractional factorial designs were used, which resulted in 18 profiles (i.e. 9 choice sets). These choice sets were blocked into Survey A and Survey B containing 4 and 5 choice sets, respectively.

Respondents were placed in a hypothetical day of leisure situation, for which three recreational options were available on that particular day. The first two options were to visit either Beach A or Beach B, with beaches differing by attributes; the third option (status quo) provided the opportunity to do something else or stay at home. We followed a state-of-the-art methodological protocol [20], randomly giving each respondent one survey type. The final section of the questionnaire had a set of socio-economic and demographic variables of the respondent, including age, nationality and the household income. At the end of the questionnaire, the respondent was given a cold soft drink as a sign of appreciation for their participation.

The questionnaire was administered in Catalan, Spanish and English through a face-to-face interview by a team of trained interviewers. The surveys were conducted in two distinct time frames, 10:00–14:00 and 15:00–19:00, on weekdays and at weekends from 14 June—15 September 2012. Only beach recreationists aged 18 years or over were interviewed. Interviewers used the shoreline as a reference line and walked ten meters straight along the shoreline or from the shoreline inland to randomly encounter each respondent. When possible, and to facilitate data collection, interviews were only conducted with beach recreationists between the shoreline and the first 30 meters, which is considered to be the useful beach area [21]. Beach recreationists were mainly approached while they were laying and sunbathing on a towel, or while they were coming out of the water and walking along the shoreline. Interviewers were identified by a badge and were responsible for explaining the specific context to the respondents, the aim of the study and the estimated duration of the survey. Two teams of interviewers were established to cover the 4 southern and the 4 northern beaches studied, respectively. The questionnaire took about 15 min for each respondent to complete.

### Economic model

As stated, we used a CE framework, which allowed individuals to select between different beach alternatives. From the economic modelling perspective, this attribute-based choice method has its theoretical grounding in Lancastrian consumer theory [22], which proposes that individual welfare is based on the consumption of goods and services, which is expressed in terms of their characteristics and respective contribution to welfare or utility. The underlying fundamental assumption is that individuals act rationally, selecting the consumption bundle, and respective characteristics, that yields the highest utility [23], [24]. In our study, we applied this economic model to analyze the behavior of the beach recreationists in order to compute the impact caused by jellyfish blooms on the tourism sector. This was presented in terms of the selection of the beach to visit. The model is described in accordance with a set of characteristics and the respondents select the beach destination according to the characteristics of the location. In formal terms, we can represent a beach recreationist as an individual *i*’s whose utility associated with the choice of a beach-alternative *j* is described as:

$$U_{ij}=V\left(Z_{j},S_{i}\right)$$ (1)

where for any respondent *i*, a given level of utility will be associated with any of the alternatives proposed *j*. The derived utility depends on the attributes of the proposed beach-scenarios (*Z*_*j*_) and respondent’s socio-economic characteristics (*S*_*i*_).

From the individual’s point of view, the selection of a beach scenario, as described in the survey, is the result of maximizing the utility; thus, the respondent’s stated choice, as reported in the survey by selection of the beach scenario, is the one that yields the highest utility. In this context, the probability of any particular alternative *j* being chosen can be expressed as:

$$P_{ij}=\frac{exp\left[V\left(Z_{ij},S_{i}\right)\right]}{\sum_{j\epsilon C}exp\left[V\left(Z_{ij,}S_{i}\right)\right]}\;\;\;\;\;\;\;\;with\;j=\;1,\;2,\;\ldots J$$ (2)

Bearing in mind the present beach attributes, we estimated the following empirical specification:

$$U_{ij}=\beta_{1}jellyfishrisk_{ij}+\;\beta_{2}waterquality_{ij}+\;\beta_{3}services_{ij}+\beta_{4}additionaltime_{ij}+\beta_{2}additionaltime_{ij}^{2}+\varepsilon_{ij}$$ (3)

In other words, the utility that the respondent *i* has from selecting beach *j* depends on the four attributes under consideration, including additional travel time, beach water quality, the services available at the beach, as well as the risk of a jellyfish bloom.

The indirect utility function was constructed to include all attributes that defined the choice elections, which contained a trade-off between travelling time and different beach characteristics. Furthermore, non-linear effects were explored. In particular, we tested for the possibility that additional travel time affected the choice behaviour in a non-linear way. In other words, we tested empirically that the marginal impact of this extra travelling time characteristic was not constant.

Bearing in mind the respondents’ answers to the CE survey, we estimated the parameters *β*_*s*_. These parameters are unknown to the economist. Therefore, we proposed estimating them exploring the use of a conditional logit model [23]. The conditional logit model (CL) assumes the independence of the irrelevant alternatives (IIA) property, which states that the ratio of choice probabilities between two alternatives in a choice set is unaffected by the other alternatives that are available in the choice and the levels of the attributes of the other alternatives. In many alternatives this is a useful property.

After estimating the baseline conditional logit model (CL), we extend our empirical section by estimating a random parameters model (RPL), given that initially we are assuming that individuals’ preferences are homogenous, while in fact it is more logical to expect that their preferences are heterogeneous. The RPL model assumes that the functional form of utility and arguments are common among individuals within the sample, but that the parameters vary (are random) between individuals.

## Results

### Data description

We received 644 completed questionnaires by respondents with an average age of 42 years. Tourists in Catalonia constituted about 57% of our sample and reported planning to stay about 16 days on holidays at the coast. In addition, international tourists represented about 24% of the respondents (Table 3).

**Table 3:** Characteristics of respondents.

Description	Mean	Std. Dev.
Male	21.8	41.3
Respondent planned to stay at this beach < half a day	72.6	44.6
Respondent planned to stay at this beach half a day	21.0	40.8
Respondent planned to stay at this beach entire day	06.4	24.4
Respondent came to the beach on foot or bicycle	47.4	49.9
Respondent came to the beach by car or by motorbike	39.0	48.8
Respondent came to the beach by public transport	13.6	34.3
Respondent has been stung by a jellyfish	21.7	41.2
Respondent knows someone who has been stung by a jellyfish	17.2	37.7
Respondent has not been stung and does not know anyone who has been stung	61.1	48.8
Respondent has his/her primary residence in this place	43.7	49.6
Respondent is international	23.6	42.4
Respondent lives in Spain	17.8	38.3
Respondent lives in Catalonia	14.9	12.3
Respondent has above high school; 0 otherwise	49.6	50.0
Length of stay (days)	15.9	24.6
Age of respondent (years)	42.7	13.5
Respondent has a job; 0 otherwise	72.2	44.8
Respondent’s household income is below 2000€	36.7	48.2
Respondent’s household income is between 2000€-4000€	44.4	49.7
Respondent’s household income is above 4000€	18.9	39.2
Time taken to reach the beach (min)	21.3	24.4

Variables are presented as percentages (%) over total sample (N = 644).

Three quarters of respondents planned to stay at the beach less than half a day, while 21% planned to stay at the beach half a day and 6% the whole day. This means that the consumption of beach recreation opportunities is concentrated in a couple of hours, and the median respondent spends less than half a day at the beach. About half of the respondents came to the beach on foot or bicycle; 39% used a car and 13% public transportation. The average time taken to reach the beach was around 22 minutes. We also observed a wide distribution of travelling time, ranging from about 5 minutes (first quartile) to 3 hours (fourth quartile). In addition, about 61% of the respondents reported that they had never been stung by a jellyfish and did not know anyone who has been stung. About 22% of the respondents reported that they had been stung by a jellyfish and 17% knew someone who had been stung. Finally, the median respondent had an education above high school level, most had a job and a household income between 2,000 € and 4,000 € per month.

### Estimation results

Estimation of the main effects model was reported in Eq 3. Model I refers to the main effects model, only considering the direct effects of the characteristics of the choice of the beach scenario. We interpreted this as our baseline model specification. All coefficients carried the expected signs and were statistically significant (Table 4).

**Table 4:** Estimation results: CLogit specifications.

	Model I	Model II	Model III	Model IV
Variable	Coefficient	Coefficient	Coefficient	Coefficient
Jellyfish risk	-0.349***	-0.374***	-0.400***	-0.367***
Water quality	0.730***	0.737***	0.738***	0.733***
Services	0.409***	0.400***	0.401***	0.410***
Additional time	0.079***	0.081***	0.081***	0.078***
Additional time2	-0.001***	-0.001***	-0.001***	-0.001***
Jellyfish risk * De Blanes	—	0.310***	0.312**	—
Jellyfish risk * Stung	—	—	0.111	—
Jellyfish risk * Resident	—	—	—	0.054
	*Scale Parameter*			
Services				
Water quality				
Log-likelihood	-1,970.409	-1,967.313	-1,966.448	-1,961.602
AIC	3,950.800	3,946.600	3,946.900	3,935.200
N	2892	2892	2892	2892

*** statistically significant at 99%,

** statistically significant at 90%

In particular, we observed that the estimated coefficient with respect to jellyfish blooms was negative. This means that a beach scenario described in the questionnaire with a profile of ≤2 days per week of jellyfish blooms (low risk) is associated with generating a higher utility to the beachgoer when compared to another beach scenario that is characterized with a profile of ≥5 days a week (high risk), *ceteris paribus*. Estimation results showed that all of the beach scenarios that are characterized by higher risk of a jellyfish bloom have a lower probability than the one which was chosen; the reduction of the probability is estimated to be 34.5%. In contrast, increases in the water quality and the range of services provided at the beach increased the probability of choice by 73% and 40.9% respectively. Thus, we inferred that the most relevant characteristic to explain the respondent’s choice was the water quality, followed by surrounding services/infrastructure and then by lower risks of jellyfish blooms. These estimates were robust across all models.

In addition, we estimated the marginal impact of the characteristics of the beach and the respondent’s on their stated choices. In particular, we assessed the impact of being at the beach in Blanes, which had the lowest jellyfish risk profile, i.e. this beach shows the lowest register of jellyfish blooms in the Catalonia coastline as recorded from 2006 to 2010. This effect was captured by the variable Jellyfish risk * Blanes in Model II and Model III (see Table 4). The estimation results reiterate the main results provided by Model I, including signal and magnitude estimates of the impact of the variables “jellyfish risk”, “water quality”, and “services”. For example, estimation results from Model II and III showed that, on average, the increased risk of jellyfish bloom reduces the probability of the choice of the beach by 37.4% to 40.0%, respectively. However, this marginal impact of this effect when valued at the beach Blanes is much lower; estimated to be 6.4% (= 37.4%− 31.0%) and 8.8% (= 40.0% − 31.2%), respectively for Model II and Model III. In other words, estimation results showed that the respondents that chose the beach Blanes have a structure of preferences for which the risk of jellyfish is not as important, when compared to the average respondent. This may be due to several reasons, including the fact that actual low-risky conditions may impact stated preferences. These results may indicate that these respondents have already adapted to the risk of jellyfish blooms by selecting the beach with the lowest risk profile.

In addition, we assessed the impact of being stung on the stated choices. This effect was captured by the variable Jellyfish risk * Stung in Model III (Table 4). This model showed that an increase in the jellyfish risk reduces the probability of the choice of the beach by 28.9% (= 40.0% − 11.1%), among the sub-sample of respondents who reported being stung before. However, this marginal impact was not statistically significant at the 90% confidence interval. In other words, for the current sample, the empirical evidence does not support a statistically difference of risk profiles among the two respondents segments: stung vs. non stung. The impact of reduction of jellyfish risk on beach behavior is not evaluated statistical different among these two segments.

Furthermore, we also evaluated the impact of being a resident in beach preferences. This effect was captured by the variable Jellyfish risk * Resident in Model IV (Table 4). Model IV showed that residents reported a slightly lower risk aversion to jellyfish blooms, but again this difference was not statistically significant. Models V-VIII (Table 5) provide the RPL estimates. These estimates are quite comparable to those CLogit results, although these latest result relax the fulfilment of the IIA property. Table 5 estimates reaffirm those provided in Table 4.

**Table 5:** Estimation results: RPL specifications.

	Model V	Model VI	Model VII	Model VIII
Variable	Coefficient	Coefficient	Coefficient	Coefficient
Means for random parameters				
Services	0.463***	0.452***	0.454***	0.468***
Water quality	0.77***	0.778***	0.779***	0.792***
Non-random coefficients				
Jellyfish risk	-0.414***	-0.446***	-0.476***	-0.437***
Additional time	0.099***	0.102***	0.101***	0.098***
Additional time^2	-0.002***	-0.002***	-0.002***	-0.002***
Jellyfish-risk*Blanes		0.365**	0.366**	—
Jellyfish risk*stung		—	0.126	—
Jellyfishrisk*Resident		—		0.060
Scale Parameters				
Services	0.127	0.123	0.1214	0.132
Water quality	0.427	0.432	0.4330	0.427
N =	2892	2892.000	2892.000	2892.000
Log-likelihood	-1945.681	-1941.832	-1940.820	-1933.342
AIC	3905.14	3899.700	3899.600	3882.700

*** statistically significant at 99%,

** statistically significant at 90%

Finally, in this study the empirical estimates on travel time did not reject the presence a non-linear effect of travel time on utility. As we can see from Models I to IV, the first additional minutes of travel time were associated with a utility gain and increased the probability of choice. We interpreted this result as indicating that the travel time to reach the beach, which was seen mainly in trips by bicycle and on foot, was associated with a positive impact in the utility by the respondent. In other words, the respondent enjoyed the time spent to reach the beach. One of the first illustrations of this phenomenon was presented by Walsh et al [25]. They developed and applied a statistical procedure to estimate a demand function for the recreation activity of pleasure driving or sightseeing by car on scenic river highways in the Rocky Mountains.

In our present analysis, the consumptive value of travel time was positive until a certain point. Mathematically, this point is computed from the RLP (Model V) equation as:

$$\frac{dU}{dt}=\frac{d\left(0.099t-0.002t^{2}\right)}{dt}=0.099-0.004t=0$$ (4)

In our study, this occurred when *t* = 24.75 minutes. Thus, after 24.75 minutes any additional travelling time produced a negative impact on utility. After this point, respondents considered that reaching the beach reduced the pleasure of travelling. As may be seen, this non-linearity is also robust across specifications.

The well-being estimates were obtained by substituting specific values of the distribution of the variable travelled time into the utility function. The traditional Hanemann [26] formula was used to compute willingness to travel (WTT) estimates. In particular, WTT for beach improvements with respect to *k* attribute was computed as:

$$WTT_{k}=\frac{\frac{dU}{dk}}{\frac{dU}{dt}},\text{ evaluated at }t\;=\;T.$$ (5)

This allowed us to compute the relative changes on the respective valuations of attributes with respect to the time travelled to the beach for various segments. Thus, respondents were willing to incur different additional travel times for the different beach consumption patterns described in the survey, depending on their reported travel time. The valuation of the attributes for the entire sample at the average time used to travel to the beach $\overset{-}{T}$ anchors all the mean WTT estimates. According to our estimates, the most valuable attribute was water quality; in particular, beach recreationists were willing to travel, on average, about 8 min additional to find a beach with higher water transparency (Table 6). With the same line of reasoning, we can estimate that beach recreationists were willing to travel about 4.5 min extra to find a beach with additional services, including a play area for children and a first aid centre. The importance of the risk of jellyfish blooms was also significant in terms of explaining respondent’s behaviour with respect to beach selection; beach recreationists were willing to travel 3.8 min more per trip to go to a beach with ≤2 days per week of jellyfish blooms (low risk) rather than one with jellyfish blooms more than 5 days a week (high risk).

**Table 6:** Valuation of the selected beach attributes expressed as additional time to travel in minutes.

Willingness to travel estimates (full sample)					
Variable	Estimate*	Std. Err.	P |z|>Z*	95% Confidence Interval	
Jellyfish risk avoidance	3.81	0.890	0.000	-5.553	-2.066
Water quality	7.98	1.773	0.000	4.500	11.450
Services	4.47	1.104	0.000	2.309	6.635

*estimates are presented as absolute values (in minutes)

However, these general results are not used for the valuation of the negative externalities carried out by jellyfish blooms. For that purpose, we take into account only the subsample that makes trade-offs between the various beach attributes. In particular, keeping in consideration this sub-sample, and evaluating the WTT estimate at the mean time used by these individuals(T = 35 m), we find that the mean WTT to avoid jellyfish blooms is 10.10 min (substituting directly into (5)). Given that the time travelled has an opportunity cost in terms of foregone income, and given that our sample indicated that their average household income per hour was €19.23 for 2012, the additional travel time a beachgoer was willing to incur to move to a beach with the same characteristics but with lower risk of jellyfish bloom equated to €3.20 (equivalent income gained in 10.10 min). We should, however, acknowledge that only about 50% of the sample make such trade-offs, giving that they face trips over 25 minutes to access the beach.

## Discussion

### Policy Analysis

As seen from the estimation results, the risk of jellyfish blooms plays an important role in explaining individual behavior with respect to the consumption of beach recreation opportunities, including the choice of beaches. Nevertheless, this characteristic, or driving force behind beach recreation consumption, was not the most important one. Estimation results ranked the improvement of the water quality as first; therefore it is interpreted as the most important factor when choosing a beach. The estimation results also informed us that reduction of risk of jellyfish blooms ranked as important as the improvement of beach infrastructure, including the provision of additional services, such as playgrounds for children.

Furthermore, we are also able to monetize the lower risk of jellyfish blooms by using the concept of value of time. Our sample results indicated that the average household income per hour of our sample population was €19.23. The additional travel time an individual (who travels more than 25 min) was willing to incur to move to a beach with the same characteristics but with lower risk of jellyfish bloom equated to 10.1 additional minutes, which is equivalent to €3.20.

Because the sampling design of this study guaranteed the regional representativeness of the holidaymakers in this area, we can scale up this monetary value to the regional level. The 2012 tourist statistics released by the Catalan government showed a total of 263.7 millions trips to the beach per year by all holidaymakers (local, domestic and international) in Catalonia. We calculated that the aggregated wellbeing gains associated with a reduction of jellyfish blooms in this area would be around €422.60 million annually [263.7*0.5 (trips)×(€3.20)], corresponding to approximately 11.95% of the tourism expenditure of the Catalan population in 2012. In this case, this significant value shows that preventive policy measures (such as current airplane surveillance) pass a cost benefit analysis providing their corresponding implementation costs are below these cost figures.

All in all, one can argue that from a policy perspective there is significant social relevance for the investment of public resources in mechanisms that deal with managing jellyfish blooms, including daily reports informing users about the presence or absence of a jellyfish bloom at each beach. An example of this type of public policy mechanism is the iMedJelly application, available for free at the App Store and Google Playstore [27], which provides daily observations on the status of the Catalan beaches that includes information on the presence of jellyfish blooms. Informational and public awareness campaigns of this kind are useful for providing public jellyfish reports and exploring the use of new technologies, such as mobile phones, internet and other social media applications, which can provide immediate and real-time information. These campaigns may help to prevent the stigmatization of certain beaches and jellyfish species by raising awareness and knowledge of these species among beach recreationists and the general public. According to the scientific community, this may be the most effective policy instrument for responding to jellyfish blooms.

## Conclusions

In this study we conducted a survey of coastal holidaymakers in Catalonia in order to understand their preferences when choosing from between several beaches that provided different recreational opportunities, with different possibilities of encountering jellyfish blooms. From this exercise, we computed the number of minutes that respondents were willing to travel to find or avoid certain specific beach characteristics. Having ascertained the number of minutes that respondents were willing to travel for each beach characteristic, we expressed this value in monetary terms from their mean annual household income. On average, each holidaymaker who was dissatisfied with the amount of travelling time was willing to pay €3.20 per trip to the beach to go to a beach with ≤2 days per week of jellyfish blooms (low risk) rather than one with jellyfish more than 5 days a week (high risk). Thus, significant welfare gain associated with the reduction of jellyfish blooms also shows that preventive and adaptive jellyfish measures pass a cost benefit analysis, providing their corresponding implementation costs are below €422.60 million per year. Needless to say, this pioneering study calls for further socio-economic investigation, including the mapping and distribution of the impacts on well-being, and discussion and evaluation of other alternative policy measures such as catching jellyfish and putting nets in place, which are currently under development and experimentation.

## Supporting Information

S1 Survey (DOCX)
