# Supplementary material for: Analyzing Beach Recreationists’ Preferences for the Reduction of Jellyfish Blooms: Economic Results from a Stated-Choice Experiment in Catalonia, Spain
Source: PLoS One. 2015 Jun 8;10(6):e0126681. doi: 10.1371/journal.pone.0126681 (PMC4459978; doi:10.1371/journal.pone.0126681)
Supplement: S1 Survey — (DOCX) [file pone.0126681.s001.docx]

S1: **Survey**

English version (also available in Catalan and Spanish)

Good-morning/good-afternoon. My name is [say your name] and I am working on Marine Sciences at the CSIC/LEITAT, Barcelona. I would like to ask you some questions regarding your attitudes and opinions on marine environmental quality and recreational behaviour on the Catalonian beaches. This is an anonymous interview and will take less than 10 minutes of your time.

We appreciate your participation very much, so we would like to thank you for it. However, we cannot offer you any monetary reward; instead we would like to offer you a soft drink to refresh yourself after the survey. May we start with this survey? Yes/No

1. Recreational data [do not read this]

***Please think carefully about each question and give your best answer. There are no right or wrong answers, only personal answers.***

**1.1** How many persons are in your group (including yourself?)

**1.2** During the **last summer season** (from June to September of 2011), how many times did you come to **this** beach?

**1.3.1** During **this summer season** (from June to September of 2012), how many times have you come to **this** beach?

**1.3.2** How many more times have you planned to go to **this** beach?

**1.4** How important are these activities/factors to you to chose to **this** beach?

**1.5** Which of the following nautical sports have you ever practiced in **this** beach?

**1.6** How long do you plan to stay at **this** beach **today**?

1. Travel Cost Expenditures Data [do not read this]

**2.1** Where are you staying?

**In case he/she is staying in his first residence/home, skip to section C3.**

**In case he/she stays in another kind of accommodation, ask for its’ typology and in case it’s a hotel/camping…etc, ask for its’ name and location.**

**2.2** How many nights are you going to stay?

**2.3** How much did it cost for you to stay in the Catalan coast **per person** with regard to:

Now we are going to talk about your stay at **this** beach only during the day of **today**:

**2.4** How did you travel from the hotel/home to the beach **today**?

**In case he/she answers by car or motorbike: how much have you spend on tolls and parking to and from your origin place?**

**In case he/she answers by public transport:** how much did the ticket cost you?

**2.5** How much time have you spent to arrive to **this** beach **today**?

**2.6** Did you rent any equipment at the beach (sun-umbrella, sun bed, windbreak, boat, fishing material, etc.), paid any permits (anchoring, etc) or spend any money (e.g. meals) at **this** beach?

**In case he/she answers yes: how much have you spent on it?**

1. Socio-economic impact of a jellyfish bloom [do not read this]

**3.1** Have you or someone you know ever been stung by a jellyfish?

**In case he/she answers NO, skip to section E.**

**In case he/she answers YES but in another beach, ask for the name of the beach.**

**In case he/she or the person who knows, has been stung several times, ask to answer the questions regarding to the last time he/she/the other person was stung.**

**3.2** How many times have you have been stung by a jellyfish?

**3.3** How did you treat the pain?

**In case you went to the first aid station/physician/family doctor: how many hours/day did you spend?**

**In case you went to the pharmacy: how much time and money did you spend?**

**3.4** Did you lose work time as a result of the sting?

**In case he/she answers YES, ask for many hours.**

**3.5** Were you able to identify the species of jellyfish that stung you? **[Please show CSIC identification of the different jellyfish species]**

1. Contingent Behaviour: impact of building of an offshore wind farm [do not read this]

I would like to ask you how your use of this beach may change with the building of an offshore wind farm for next year. The proposed changes are described in CARD 1. [Please show CARD 1]

**4.1** Suppose that next year the change described in this card takes place in this beach. Would you like it?

**4.2** Would you change the number of trips you would do to this beach over the next years´ summer season?

**In case that he/she would reduce the number of trips, ask how many fewer trips he/she would do.**

**In case he/she would not come to that beach again and that he/she would do something else, ask what she/he would do.**

1. Choice experiment

Now, imagine you are planning to go to the beach. I’m going to show you some cards in which you will see two profiles of beaches according to different attributes and characteristics. Could you choose from between the two possibilities shown? Please take into account that if neither of the two possibilities shown fits with your preferences and wishes, you can also decide not to go to the beach. [Please show CARD A]

1. Socio-economic demographic questions [do not read this]

*We are finishing the questionnaire. Before, I would need to ask you some additional questions about you.*

**6.1** Year of birth

**6.2** Place of birth

**6.3** Where do you live? Ask for postal code if he/she knows it.

**6.4** What kind of form of education have you got?

**6.5** What is your job? **In case the person is unemployed, ask for his/her last job.**

**6.6** How many people live in your household?

**6.7** In which interval is the **after tax income** of your household **per month**? (if you do not share your income with the people you are living with, could you please tell me an approximate interval of your own income? **[Wait for a spontaneous answer]** Take into consideration if the respondent is a student without job or he/she is unemployed without receiving unemployment benefits.

1. FEEDBACK

Thank you very much for your time [**hand him/her the soft drink**]**,** We hope the survey was of interest to your. Would you like to receive further information and the results of this survey? If so, could you please give us your email address?
